# How big is too big? A qualitative study of discretionary food portion size norms among Australian consumers

**DOI:** 10.1017/S1368980024001964

**Published:** 2024-10-24

**Authors:** Qingzhou Liu, Leanne Wang, Margaret Allman-Farinelli, Anna Rangan

**Affiliations:** 1 School of Life and Environmental Sciences, Faculty of Science, The University of Sydney, Sydney, NSW 2006, Australia; 2 Charles Perkins Centre, The University of Sydney, Sydney, NSW 2006, Australia; 3 Discipline of Nutrition and Dietetics, Susan Wakil School of Nursing and Midwifery, Faculty of Medicine and Health, The University of Sydney, Sydney, NSW 2006, Australia

**Keywords:** Consumer behaviour, Norm, Portion size, Discretionary food

## Abstract

**Objective:**

The high availability of energy-dense nutrient-poor discretionary foods in large serving and package sizes may have shifted portion size norms (described as a typical perception of how much people choose to eat from a given food at a single eating occasion) towards larger sizes. Few public health recommendations exist around appropriate discretionary food portion sizes. This qualitative study aimed to explore the underlying rationale of portion size norms of discretionary foods among Australian adults 18–65 years.

**Design:**

Four focus group sessions were conducted. Collected data were analysed using inductive thematic analysis.

**Setting:**

Focus groups were held online via Zoom between September and October 2023.

**Participants:**

Thirty-four participants were recruited in the study (mean age 38 years, 19 females).

**Results:**

The key themes raised from inductive analysis were personal factors, eating context factors and food environment factors relevant to the portion size norms. A framework was established to illustrate the interaction across these themes during the conceptualisation of the norms. For serving size availability, consumers found that there were limited serving size choices when making portion size selections and lacked the knowledge and skills in portion control.

**Conclusions:**

These findings highlight the need to make positive changes to the current food environment and develop relevant public health guidelines around appropriate portion sizes to promote healthier portion size norms and enable better portion control.

Portion size norms, described as a typical perception of how much people choose to eat from a given food at a single eating occasion^(1)^, play an important role in portion size selection and dietary behaviours^(2,3)^. The ubiquity of large servings and packages may act as normative cues that shift portion size norms towards larger sizes^(4–6)^, a phenomenon commonly known as ‘portion distortion’^(1,4,5)^. It can lead to consumers regarding larger serving sizes as their new ‘normal’ and smaller serving sizes as ‘less than acceptable’, resulting in overconsumption over food and energy intake^(4,7)^. This may be a potential explanation for the significant increase in typical portion sizes over the past 20 years in the Australian population in particular for energy-dense nutrient-poor discretionary foods (defined as ‘foods that are high in added sugar, added salt, saturated fat and/or alcohol and are not a necessary part of healthy diets’ such as lollies, chocolates, packaged crisps, biscuits, cakes, soft drinks and/or fast foods)^(8–10)^. Nutrition education interventions alone were found to have minimal impact on reducing the amount of food consumed when people are exposed to large serving sizes^(11,12)^. Stronger public health measures are needed to promote healthier portion size norms. For instance, clear public health guidelines around appropriate discretionary food portion sizes and a multi-sector approach would be required to make changes to the food environment (described as the consumer interface within a food system that encompasses the availability, affordability, convenience, quality and promotion and sustainability of food)^(4,13,14)^.

The development of public health guidelines and campaigns aimed at reducing discretionary food portion sizes is hindered by the current lack of understanding around portion size norms and cognitive determinants of portion size selections^(1,13)^. The complex nature of food choices and portion size norms have been recognised, as they are embedded within the broad sociocultural environment and dependent on various individual factors, such as gender, weight status and dietary restraint, and contextual factors, such as peer influence and context of eating^(15–17)^. For example, the perceptions of appropriate portion sizes appeared to be associated with social expectations of how much should be consumed, and people tended to behave in a socially desirable manner to avoid disapproval from others^(2,15)^. Previous studies have shown that individuals considered a range of serving sizes as ‘normal’,^(15,18)^ and reducing serving sizes to the lower end of this ‘norm range’ would reduce consumption unconsciously without triggering compensatory eating^(18)^. Learnings from the unsuccessful sugary drink cap rule in New York City (serving sizes of all sugary drinks restricted to less than 500 ml) revealed that implementing large reductions and restrictions on serving sizes is challenging as a result of opposition from the general public, retailers and food industry^(19)^.

Portion size norms have been assessed using quantitative measures such as a judgement task of the normality of displayed portion size options, self-selected normal and appropriate portion sizes using real foods or food images, as well as an estimation of the number of ‘typical’ portion sizes contained in a large container^(18,20,21)^. From a public health perspective, however, it remains unclear how portion size norms are conceptualised, how consumers perceive the current serving size options available in the food environment and whether the options align with their preferences. Therefore, this study aimed to explore the underlying rationale of discretionary food portion size norms among the Australian adult population aged 18–65 years, examine differences between normal *v*. appropriate portion size norms and investigate consumers’ opinions on the availability of serving size options in the current food environment.

## Methods

### Study design

This qualitative study was conducted and reported in accordance with the Consolidated Criteria for Reporting Qualitative research checklist^(22)^. Adult consumers aged between 18 and 65 years were recruited via social media (Facebook, CA, USA, 2023) to participate in a one-hour semi-structured online focus group related to ‘food portion sizes’. Participants who met the following criteria were considered eligible for this study: living in Australia, aged between 18 and 65 years, no current or previous diagnosis of an eating disorder and regular consumers of discretionary foods (that is, consume discretionary foods more than three times per week).

The screening questionnaire was developed using Qualtrics (Provo, UT, USA, 2022). An embedded function was used to detect bots and repeated attempts, and screener results were manually checked to ensure accuracy. In the screener, regular consumers of discretionary foods were identified via the question, ‘How many times would you consume discretionary foods such as lollies, chocolates, packaged crisps, biscuits, cakes, soft drinks and/or fast foods in an average week?’. Participants could select either ‘less than three times a week’ or ‘three or more times a week’ as their response. Participants were unaware of the eligibility criteria before they completed the screener.

Focus groups were held online via Zoom version 5.16.10 (Zoom Video Communications, CA, USA, 2023) to enable recruitment of consumers living in both metro and rural areas. The use of online videoconferencing tools for qualitative data collection has gained popularity in recent years and is considered a valuable alternative to traditional in-person set-ups to recruit more geographically diverse samples and reduce participant burden (for example, less travel time to attend the study)^(23,24)^.

### Theoretical framework and question guide development

This study was specific to discretionary foods, defined as ‘foods that are high in energy, added sugar, added salt and/or alcohol, and are not a necessary part of healthy diets’^(10)^. Foods from the five food groups (grain foods, fruit, vegetables and legumes/beans, meat and/or alternatives, milk and/or alternatives) were not within the scope of the current study^(10)^. Questions used to guide the focus group discussions (Table 1) were informed by the literature on portion size selections and norms^(1,15,25)^. Three different eating contexts were included; home, coffee shops (a room or building where coffee and light refreshments are served)^(26)^ and fast-food outlets. A series of questions were developed to investigate participants’ impressions of serving size options in these settings, how they decided on their normal and appropriate portion sizes and potential factors influencing their portion size norms and decisions.

**Table 1.** Questions used to guide focus group discussions

Eating contexts	Questions
Home	Think about a typical day at home; How do you normally decide on the size of cake or pastry you would have as a between-meal snack?
	What kind of packaged foods (for example, packaged crisps, sugary or diet soft drinks) do you have at home?
	Why did you choose these package sizes?
	How do you select your normal amount to consume in one sitting?
	How do you decide on the amount that you would consider to be appropriate to consume in one sitting?
	Do you consider your normal and appropriate portion sizes to be the same or different? Why? (A sliding scale with different serving size options for multiple common discretionary foods was shown)
Coffee shops	Think about a recent trip to a coffee shop; What is your impression of the sizes of the pieces of cakes and/or pastries available?
	What, if anything, influences your choice of serving sizes (to purchase) and portion sizes (to consume)?
Fast food outlets	Think about a recent trip to a chain fast-food outlet; What is your impression of the sizes of burgers available?
	What, if anything, influences your choice of serving sizes (to purchase) and portion sizes (to consume)?

We acknowledge that there is currently no conceptual framework for portion size norms and selections. The conceptual model of food choices was used as a basis to develop questions as it recognises the complexity of eating behaviours^(27)^. This model describes the multi-level components involved in the decision-making process of food choices, including factors at individual and larger societal food-system levels that interact with one another and lead to the point of food choice^(27)^. There was a focus on the impact of both personal and external environmental factors and the interaction between these factors during the conceptualisation of portion size norms and the decision-making process of food choices. The question guide was pilot tested multiple times using a convenience sample of the target population (*n* 14).

### Participants and recruitment

We used the concept of information power to guide sample size assessment, which proposes that the more information relevant to a study a sample holds, the lower the number of participants required^(28,29)^. This concept has been widely used in qualitative studies in health science where the focus was to gather salient information to answer research questions rather than to generate a complete list of all possibilities^(28,29)^. The sample size of 32–40 participants, with eight to ten participants per group, was considered sufficient to gather rich information to answer our research questions.

Potential participants were recruited through online advertisements with a link directing participants to a screener (Qualtrics, UT, USA, 2022) to assess their eligibility for the study. The study was approved by The University of Sydney Human Research Ethics Committee (2023/089). Written consent for study participation and contact details (email address) were obtained through the screening questionnaire. Eligible participants were contacted by a member of the research team to schedule a focus group session time. Participants received a voucher ($15 Australian dollars supermarket gift card) as reimbursement for their time after completing the focus group.

### Focus group procedure

The four online focus groups took place between September and October 2023. All focus groups were facilitated by one primary moderator, a female Accredited Practicing Dietitian (Q.L.) and observed by a second moderator who attended the focus groups on a rotating basis (A.R. and L.W., experienced Accredited Practicing Dietitians). Participants were required to join the online group session in a quiet room and turn their electronic devices on mute to minimise interruptions. The study aims and definition of discretionary foods were introduced at the beginning of each session. The focus group discussion was facilitated using the pre-determined question guide. Fieldnotes were taken by the secondary moderator during each focus group to capture non-verbal expressions and other contextual details. Members of the research team had no personal relationships with the participants. Reassurance was provided at the beginning and throughout each session to emphasise that there were no right or wrong answers, and neutral words were consistently used to minimise researcher bias^(30)^. Reflective practice was performed after each focus group through memos, group meetings and discussions to ensure the non-judgemental nature of the study^(30,31)^.

Basic demographic information including age, gender that they most identified with, postcode of home address, email address, physical activity level, cooking confidence and the average number of times per week discretionary foods were consumed were collected at the end of each session using a short online survey (Qualtrics, Provo, UT, USA, 2022). The physical activity level was estimated based on the intensity and frequency of usual exercise^(32)^. Cooking confidence was measured using a five-point validated Likert scale to rate the following four statements: ‘I can cook a nutritious meal’, ‘I can cook a meal in a short amount of time’, ‘I can cook without spending a lot of money’ and ‘I can follow a recipe’)^(33)^. A total cooking confidence score of < 16 was classified as low and a score of 16–20 as high. The socio-economic status was estimated using the socio-economic indices for areas^(34)^; self-reported postcodes were assigned into deciles (0–10), with deciles 1–5 classified as lower socio-economic status and deciles 6–10 as higher socio-economic status.

All sessions were video- and audio-recorded using a built-in function on Zoom. Analysis was performed using audio recordings only; video recordings were deleted immediately after completion of each session.

### Data analysis

Audio recordings were transcribed verbatim using the auto-transcription function on Microsoft Word version 2310 (Microsoft Corporation). Transcripts were manually checked by the primary moderator and discussed with the secondary moderators to ensure accuracy. Transcripts were not shared with study participants.

Data coding was performed using the NVivo software package for qualitative data analysis version 14 (QSR International). An inductive thematic analysis approach was used to identify patterns of themes within the data^(35)^. Data analysis was performed iteratively throughout the data generation process to ensure analytic reflexivity^(31,36)^. Braun and Clarke’s six-phase process for conducting thematic analysis was followed^(35,37)^. The initial phase of data familiarisation involved the researcher (Q.L.) reading through each transcript multiple times. Initial coding was generated by one researcher (Q.L.) and crossed checked by a second researcher (A.R.). The code list was then refined and reviewed; codes sharing relevant concepts were collapsed into broader patterns of meaning for the development of potential themes. Potential themes and sub-themes were further reviewed, and a preliminary framework was developed through discussions among the research team. The associations between components and factors within the framework were repeatedly revised and applied to the body of data until an agreement was reached.

## Results

### Participant characteristics

A total of eighty-six participants passed the online screener and were invited to partake in the study. Participants were excluded (*n* 52) if they did not reply to the invitation email (*n* 28), were unable to attend the focus group at the scheduled time (*n* 23) or did not provide a valid email address in the screening questionnaire (*n* 1).

Thirty-four participants completed the focus groups (mean age 38 (sd 14) years, 19 females) and were included in the analysis. The number of participants in each focus group varied between seven and ten. The majority of participants lived in a postcode categorised as higher socio-economic status (*n* 28), reported a light to moderately active level of physical activity (*n* 29) and reported high cooking confidence (*n* 21). Details of participant characteristics are presented in Table 2.

**Table 2.** Participant characteristics (*n* 34)

Age, years	
Mean	38
sd	14
Gender, females, *n*	19
Education level, *n*	
High school or below	4
Diploma or equivalent	9
University	21
Socioeconomics (SES), *n*	
High	28
Low	6
Location, *n*	
New South Wales	16
Victoria	13
Queensland	2
Western Australia	2
Tasmania	1
Physical activity level, *n*	
Sedentary	2
Lightly to moderately active	29
Very to extremely active	3
Average number of times per week discretionary food is consumed, *n*	
1–3 times	16
4–6 times	14
7 times of more	4
Cooking confidence, *n*	
Low	13
High	21

### Overview of findings

**Figure 1.**
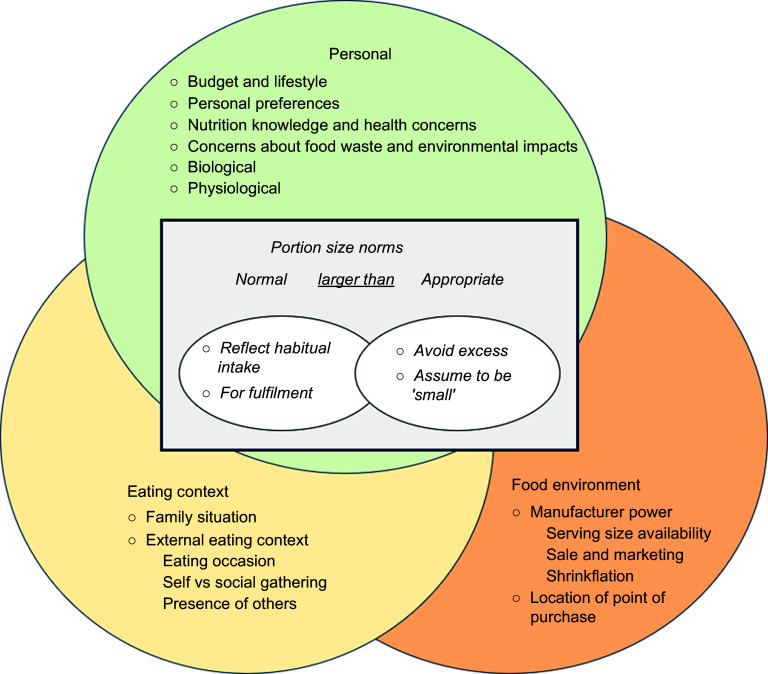
The conceptual framework of portion size norms for discretionary foods. This framework presents the interaction of personal factors, eating context, and the broader external food environment on the conceptualisation of portion size norms.

A total of fourteen themes and eighteen subthemes were raised from the inductive thematic analysis. Themes were further aggregated into personal, eating context and food environment factors related to conceptualisations of portion size norms. A framework was developed based on these findings (Fig. 1). This framework illustrates the interaction of personal factors, eating context and the broader external food environment on the conceptualisation of portion size norms. Selected quotations are present in Table 3 to describe the details of each component within the framework.

**Table 3:** Illustrative quotations of themes and sub-themes from study participants

Themes	Subthemes	Quotations
Factors influencing the portion size norms		
Personal factors		
1. Budget and lifestyle	1. Unit price	‘The first thing is price and then we have a look at what are you getting for that price.’ [54, F]
	2. Convenience	‘I take like the small packs when I go to work because it’s just like easy and convenient to grab that on the way out, but they’re definitely not satisfying at all. They’re like, really small and expensive for what they are.’ [22, F].
	3. Quality over quantity	‘We had a little French bakery near work and we actually started going there because it was a little pricier, but it was a lot better and it tended to be a much smaller amount.’ [48 M]
2. Personal preferences		‘The larger pack (crisps) and particularly when it’s the salty ones or the hotter tasting ones, could you just seem to keep eating it?’ [54, F] ‘For me, crisps, like savoury, is easier to eat, a lot more of than sweet. I find after a few bites of something sweet, I can’t eat anymore. But with the salty stuff, you can really like devour a whole packet.’ [43, F]
3. Nutrition knowledge and health concerns	4. Past experiences, family role modelling	‘There was something our parents told me as a kid, which I realised is quite clever now, which is you can have a snack like a handful. And of course, when you’re a little kid, I realise your hand is very small, so you might not get many chips.’ [48, M]
	5. Concerns about health and overconsumption	‘I don’t drink sugary or even diet drinks because they are so unhealthy and I don’t actually think that anyone should be drinking them myself, because the diet ones are full of artificial sweeteners which are carcinogenic, and the sugary ones are full of sugars.’ [60, F]
	6. Lack of understanding of recommended portion sizes	‘I mean on top of my head. I don’t know what the recommended size or you know would be.’ [47, F] ‘The back of the chips packets, they have this recommendation serving size, but I don’t know where that comes from, so I don’t follow that serve and so I was, I just think, you know, I know when I’ve had enough so that’s my serving size.’ [61, M]
4. Other concerns	7. Food waste	‘I really hate wasting food and at home I do everything to avoid it, but I do sometimes just chuck the last third (pastries take away). You just get over it. It’s all stuck to the paper and you’ve kind of had enough of the same taste so yeah, probably wouldn’t eat it all.’ [48, M]
	8. Environmental impacts of packaging waste	‘I do still generally try and buy the bigger packs (crisps), cause again per unit price. They’re better and I feel better about less packaging, right, like, just environmentally.’ [34, F]
5. Physiological and biological factors	9. Hunger	‘Generally, the sort of governing decision-making factor is how hungry I am, not how thirsty I am. So it would be like if I’m really hungry, I’ll get a large (fast food meal) and that just so happens to come with the large drink.’ [36, M]
	10. Mood	‘I mean it (portion size) depends also how I you know if I’ve had a really bad day, I might eat some more because I’m feeling depressed or whatever.’ [61, M]
	11. Gender and age	‘Most of the time when I go to the cafe and I get quite a big portion of cake, I’m just more inclined to finish it because I feel guilty not finishing it off and I feel it’s too much of a hassle to bring home. And yeah, that’s how I feel about it.’ [18, M] ‘I share them (cake or pastries) with the house(hold), I may have one or two, and then the rest will be shared or, you know, used for another day or two, because those you can usually heat up some, some of the stuff like the muffins you can freeze.’ [43, F]
Eating context factors		
6. Family situation		‘I used to not have it at all, but my boyfriend’s family has lots of soft drink and I grew up in a family with like zero soft drinks, so now I drink some.’ [24, F]
7. Other dynamic factors	12. Eating occasion, snack *v*. meal size	‘The soft bake is one portion and they’re all individually wrapped, and the biscuits come in individual wrapping with four biscuits in, which makes for a perfect snack.’ [60, F] ‘If I go to McDonald’s, for example, uh and I feel that’s not enough. I just buy like, some kind of dessert, like the apple pie or the Mcflurry ice cream, it’s just to make you a bit more full after you get that small burger, so that’s what usually happens.’ [28, M]
	13. Presence of other people – social gathering	‘I don’t buy them (soft drinks), but I will buy them if I’m having, like, a party or get together or barbecue because most of my friends will drink some kind of soft drink. So then I will buy the bigger like, well, pretty much the litre bottles.’ [43, F]
	14. Presence of other people – sharing	‘I went with another adult female friend recently to a just a suburban cafe. And we both wanted cake, but the pieces were too big, so we ended up buying one, you know, and sharing it, like splitting it in half. Because a piece of hedgehog cake that was probably like 15 centimetres long. So I was just lucky that we wanted the same one, because that way we could share it.’ [36, F]
Food environment factors		
8. Availability of serving size options	15. Restrict portion size selections	‘I feel like also a lot of cafes, more chain cafes, with their portions, they have deals. So like you get a coffee and you get a muffin, you don’t really get a choice of the different option. So like you just have to have that big muffin. That’s what I’ve noticed.’ [24, F] ‘If you’re going for a certain burger, it will come with a certain size chips and a certain size drink just for so what you think that you want for your burger, then everything else has just become the add-ons that come with it.’ [54, F]
	16. Portion sizes rely on serving sizes	‘If I’m reaching for a snack and it’s like already, like the prepackaged portions that will kind of determine like that’s just what I’ll eat unless I’m really hungry and there is like another packet or whatever then I can open that.’ [18, F] ‘The whole idea of portion size. It’s not something that you really think well, you don’t really think too much about. You just sort of, yeah, kind of just whatever they serve you. It’s not something I really think about when I buy something.’ [47, M]
9. Sale and marketing strategies		‘I also buy the 100 (gram), I think they’re about 170 gram (packaged crisps) that that kind of shared pack size, whatever’s on special that week, really.’ [49, F] ‘Sometimes there’s a psychology behind that portion sizes like you only need to add one or two dollars upgrade or order (fast foods). And even though you know that it’s a trap, then you voluntarily give into that trap because you’re getting more bang for your buck. So I tend to upgrade sometimes my meal even though I just want the small size or regular one, yeah.’ [37, M]
10. Shrinkflation		‘I think they’ve got smaller over the years. Like you open it and you’re like oh, that looks smaller than last year, but the price has gone up. I mean, that’s the same with ice cream. Packets chip packets. Everything’s got smaller, but more expensive, but I think, a lot of the food in that fast food, used to get very big sizes. Now I think it’s much smaller.’ [43, F]
11. Physical location	17. Differences between point-of-purchases	‘Just general, yeah, just general cafes. I think the mainstream cafes have got set sizes that they give you, local cafes that are not as stringent. They will cut up a slice of cake, whether it’s slightly thick or slightly thin and they’re not as regimented as the mainstream people.’ [61, M]
	18. Country differences	‘I have to say I did my undergraduate in the US because I was on a scholarship there and I went to a couple of fast-food chains there, and their food sizes are super overwhelming. So coming back to Australia’s kind of like, it’s hard because I feel like, for example, McDonald’s over there is just gigantic with all sizes and then here it’s like a lot smaller.’ [22, F] ‘I guess I’m originally not from Australia, but I just moved here like a couple of years ago. And then in my country, Indonesia, I usually got, when I ordered Big Mac, it turns out it’s it’s quite big. I mean it’s not like gigantic but it’s big. But when you when I arrived here and then tried to order the Big Mac, they are just really small.’ [28, M]
Portion size norms		
12. Norms of normal portion sizes		‘Even though the larger bottles are better value, it, I, I buy like a multi pack (soft drinks) of small sizes so the 250 mL can or the 330 mL little bottles, because I actually feel satisfied by the time I’ve had that.’ [49, F]
13. Norms of appropriate portion sizes		‘When I grew up, our parents used to divide the chips cause there’s three of us into three bowls and we had three small bowls each, and they said that’s the appropriate serving size and you’re not going to get anymore.’ [61, M] ‘I would say appropriate (portion size) is not drinking any diet soda.’ [48, M]
14. ‘Normal’ and ‘appropriate’ portion sizes distinguished		‘So I think I could be wrong, but I think yeah, like for the amount (normal portion sizes) I think I eat more than the standard amount (appropriate portion sizes), but I think for me like number four (of sliding scale for packaged crisps, corresponding to 40 g) is enough like it fills me up.’ [33, F] ‘I just said (option) six (corresponding to half pack of 170 g packaged crisps) is probably honestly what I do like at once, but (option) two (a 17 g multipack) is probably what I consider like strictly probably appropriate. So I definitely overdo like I know I overdo it, just run with it.’ [34, F]

### Personal factors

#### Budget and lifestyle

Depending on lifestyle situations, some participants appeared to be more aware of price than others when making portion size decisions. For example, a female participant in her 30s described herself as a ‘unit price shopper’. This was confirmed by another participant who mentioned that ‘the first thing was price’ when making purchase decisions (Table 3 Subtheme 1).

Discussions between purchasing multipacks for convenience *v*. purchasing larger serving sizes for better value were consistently raised in all focus groups. Larger serving and package sizes were viewed as more economically friendly. Multipacks and individual packaging were recognised as good for worksites or on-the-go as they could be finished in one eating occasion (Table 3 Subtheme 2). They were generally preferred by those who had a busy lifestyle, travelled to work and/or were not living with a large family. The quality of foods was sometimes prioritised over value for money (Table 3 Subtheme 3), with people preferring to travel to specific food outlets that offered smaller servings of foods that were better quality.

#### Personal preferences

Personal preferences in taste and flavour were acknowledged by many participants when describing their portion size norms. Participants tended to categorise themselves according to their flavour preferences, such as ‘not a sweet person’, ‘having a sweet tooth’, or ‘sweet and savoury camps’. The tendency to overeat seemed to vary by taste and flavour preferences. For instance, they recognised savoury packaged crisps were ‘a lot easier to eat more of than sweet foods’ and that they can ‘devour a whole packet’. Soft drinks in cans *v*. bottles were specifically cited as an example of taste influencing their purchasing decision and its influence overrode that of serving size. Soft drinks in cans were believed to taste better and better able to keep the ‘fizziness’. Thus, they were highly preferred and more frequently purchased than soft drinks in bottles.

#### Nutrition knowledge and health concerns

Family role modelling and childhood experiences were frequently mentioned. Participants recalled their parents teaching them about appropriate portion sizes of discretionary foods, such as ‘a handful’. Participants carried such perceptions through to adulthood, resulting in beliefs that portion sizes of discretionary foods needed to be small (Table 3 Subtheme 4).

There was general agreement that discretionary foods were ‘bad’ or ‘fattening’ and should be limited. Many expressed concerns about overconsumption and commented that large portion sizes of discretionary foods were ‘too unhealthy to eat the whole thing’ (Table 3 Subtheme 5). Participants stated that their knowledge of the unhealthiness of discretionary foods came from online sources and articles. However, no one mentioned guidelines from credible government bodies or public health organisations.

People had limited understanding of recommended portion sizes of discretionary foods and were not aware of existing dietary guidelines. On-pack serving size information on the nutrition information panel were regarded as ‘recommendations’. However, the serving size amounts were perceived to be too small and unhelpful by many participants and were not followed. Instead, they stated, ‘I know when I had enough’ (Table 3 Subtheme 6) and have developed their own strategies to determine discretionary food portion sizes according to existing nutrition knowledge and past experiences as described above.

#### Concerns about food waste and environmental impacts of packaging

Concerns around food and packaging waste consistently emerged from discussions in all focus groups. Participants acknowledged the tendency to consume more than intended, or even finishing the entire serving to avoid wasting food. Many mentioned that food wasting behaviours (that is, not finishing food or discarding leftovers) would lead to emotional consequences such as ‘regret’ and ‘guiltiness’. Depending on the family living situation, younger participants living alone or in small families revealed preferences for smaller serving size options to prevent food waste and for greater convenience. They commented that larger serving size options were ‘unpractical’ and ‘difficult to finish’. Others mentioned storage strategies such as the freezing of larger serving sizes of cakes and pastries to save for later consumption and minimise food waste.

The negative environmental impacts of packaging and plastic waste were consistently raised. Negative attitudes towards discretionary foods in multipacks were noted as they were identified as having ‘unnecessary packaging’ and ‘too much plastic waste’. Suggestions for more environmentally friendly portion control strategies included dividing larger serving sizes into smaller containers or using food sealing clips. A minority acknowledged the potential benefits of smaller multipacks for portion control and convenience.

#### Physiological and biological factors

The effects of physiological factors such as hunger and mood on portion size selection were noted. Participants spoke of the influence of hunger on the selection of fast food portion sizes when consumed as a main meal and considered the level of hunger to be the ‘governing decision-making factor’ (Table 3 Subtheme 9).

In terms of the influence of mood, people acknowledged that their portion size selections varied depending on their emotional state. Larger portion sizes were associated with a depressive mood, for example, when they have ‘had a bad day’ (Table 3 Subtheme 10).

The influences of biological factors such as gender, age and BMI on portion size norms and selections were mentioned. For example, two young males seemed to be less likely to save foods for later consumption and perceived taking away uneaten foods as ‘too much of a hassle’ (Table 3 Subtheme 11). They tended to either consume the entire portion at once or discard any leftovers. Contrastingly, middle-aged adults, especially those with kids, considered more factors when making portion size decisions, such as the value for money, potential health outcomes, as well as potential strategies for better controlling portion sizes and reducing food waste.

### Eating context factors

#### Family situation

As described in the previous section, family experiences during childhood appeared to play a role in shaping perceptions around portion sizes and nutrition-related knowledge. Associations between their current living situation and portion size selections were also raised in the focus groups. People indicated that their portion sizes heavily depended on the habitual eating behaviours of their immediate family members, such as partners and children. Examples included consuming larger portion sizes of homemade foods, consuming larger portion sizes due to finishing family members’ leftovers and smaller or larger portion size selections to accommodate the discretionary food preferences of family members.

#### External eating contexts

A clear distinction in portion sizes was identified between eating occasions, such as a snack or a main meal. Snack portion sizes were expected to be small. A person described the size of muffins at coffee shops to be ‘a lunch time meal rather than a snack’ to emphasise that the muffin serving sizes available were mostly too large (Table 3 Subtheme 12). Multipacks or individually wrapped biscuits were used as examples of preferred sizes for snacks. People expected larger portion sizes of main meals such as burgers from fast food outlets. Many participants expressed that burgers as a single food item should be ‘big enough’ to make them feel full for a reasonable period, without the need to purchase meal deals or additional side dishes.

Moreover, participants stated that discretionary foods in larger package sizes were only purchased for social events such as gathering with friends, whilst smaller serving sizes were perceived as more suitable for individual consumption.

The presence of other people was identified as another important consideration for portion size selections. When dining out in coffee shop settings in particular, multiple participants shared that they would only decide to purchase cakes or pastries in larger serving sizes when accompanied by friends or family members, so that they could ‘split’ one cake in half (Table 3 Subtheme 14)

### Food environment factors

This section describes the effects of factors in the broad food environment and the influences of external stakeholders such as government bodies and the food industry on portion size norms. Particularly, the ‘power’ of the food manufacturer and outlets consistently emerged in all focus group discussions.

#### Availability of serving size options

When commenting on the current food environment, participants found that their portion size selections were often restricted by the lack of size options. For example, the available serving sizes in coffee shops were described to be ‘too big’, ‘enormous’ and ‘unpractical’, and there were no smaller size options for particular food items such as cakes and muffins (Table 3 Subtheme 15). Participants who preferred smaller cakes or pastries experienced difficulties finding the serving size they wanted, although one person mentioned being able to select smaller sizes by switching food items. For example, purchasing a macaron instead of a cake slice, as serving size variations depended on the specific food items available at the point of purchase.

Participants expressed wanting more flexibility in serving size options to adapt to their personal preferences, rather than being limited by the default sizes. For example, people noted that food outlets set a ‘default size’ for certain food items, which were considered smaller (for burgers in fast food outlets) or larger (for cake and pastries in coffee shops) than the size they desired.

Participants seemed to be aware that their portion size norms and consumption habits relied on serving or package sizes and on-pack information, especially among those who were more conscious about portion control. For instance, the multipack was used as a stop point to help avoid overconsumption, which was perceived to be ‘dangerous’ (Table 3 Subtheme 16). However, a minority appeared to be more passively involved in the process and simply chose to ‘finish whatever was served’.

#### Sale and marketing strategies drive serving size purchase decisions

Participants paid attention to price and promotion and tended to purchase serving sizes that were more frequently on sale; for example, the 170 g packaged crisps in supermarkets and medium-sized meal deals in fast food outlets. In contrast, smaller serving size options such as multipacks were considered to ‘not go on sale’ and be ‘more expensive’ in terms of unit price. Many people considered their choice of serving sizes to be ‘manipulated’ by food manufacturers or outlets, but admitted that the temptation of purchasing discretionary foods of higher value for money was difficult to resist. Serving sizes that were more often on sale appeared to become the typical size most frequently purchased.

#### Shrinkflation

There was agreement that discretionary foods have ‘shrunk’ over time, with serving or package sizes becoming smaller but prices becoming more expensive. People noticed obvious reductions in the serving sizes of burgers from chain fast food outlets and believe they are now smaller than acceptable. Various other discretionary foods including packaged crisps, chocolates and ice creams were also mentioned as examples of shrinkflation.

The term ‘trap’ was used multiple times when people described their experiences of shrinkflation; they considered shrinkflation to be the manufacturers’ marketing strategy to promote profit.

#### Location of point of purchase

People were aware of the variations of serving size options across places and selected different point of purchases based on individual needs and size preferences. Local coffee shops were used as an example of offering ‘smaller but better quality’ options, as opposed to ‘mainstream’ chain coffee shops that had cakes and pastries in larger sizes (Table 3 Subtheme 17).

In addition, participants observed country or region differences in serving size options and availability. Serving sizes of discretionary foods in the USA and Indonesia were thought to be generally larger than the serving sizes in Australia, whilst those in Hong Kong were considered smaller. The large serving sizes of fast foods in the USA were described as ‘overwhelming’ and ‘gigantic with all sizes’ (Table 3 Subtheme 18).

### Norms of normal and appropriate portion sizes

People described their norms of ‘normal’ *v*. ‘appropriate’ portion sizes as being different. Normal portion sizes were often referred to as their habitual intake in everyday situations and relied heavily on the package or serving sizes provided. For example, participants would mention, ‘I go for a handful of chips over a larger pack’ or ‘normally half size of the pack’ (Table 3 Theme 12). Most people associated normal portion sizes with fulfillment, and they would deem to be ‘not fully satisfied’ after consuming smaller amounts. A minority described normal portion sizes as the upper limit to avoid potential negative emotions such as the feeling of excessiveness or guilt.

Participants assumed appropriate portion sizes to be small and conceptualised appropriate portion sizes as ‘how much should be eaten’. The sense of appropriateness appeared to be sourced from external information, including family conventions, online information, as well as cues on food products such as food unit size, package sizes and on-pack nutrition information (Table 3 Theme 13). The majority referred to small single unit servings or packages as the appropriate amount (for example, multipack of crisps and mini soft drink cans), with many believing that discretionary foods should not be consumed at all from a health perspective and thus the appropriate portion size should be zero. However, rather than following the norm of appropriate portion sizes, people admitted that they consciously decided to consume ‘slightly larger’ amounts for satisfaction. Difficulty to resist the temptation or craving for palatable foods was frequently mentioned to explain the underlying conceptualisation process.

## Discussion

This study aimed to explore the underlying rationale of discretionary food portion size norms among Australian consumers. A conceptual framework was developed from the themes and subthemes that emerged from the focus groups. This framework provides a holistic overview and illustrates the complex process involved in the conceptualisation of portion size norms for discretionary foods, influenced by personal factors, the context of eating and food environment factors interacting with one another.

Within the framework, personal factors including biological and physiological features, budget and lifestyle, personal preferences, nutrition knowledge and related concerns all impacted on the individual’s portion size norms. Previous quantitative studies that focused on biological and physiological factors similarly observed that portion size norms differed by individual characteristics^(17,38,39)^; males and individuals with overweight and obesity reported larger self-selected normal portion sizes. The themes that emerged from the present study were specifically related to portion size norms and selections, rather than general food choices. Participants identified the major role of their personal preferences, budget and lifestyle factors on portion size norms. The importance of these factors varied across individuals; some consumers may value a larger quantity, and others value higher quality, better taste, or convenience. In particular, many participants admitted that their purchasing decisions were driven by unit price with larger serving sizes being preferred over smaller ones. However, the opposite was also observed among participants who had busier lifestyles, where convenience tended to be prioritised, and therefore, smaller serving sizes that were more easily transported and able to be finished in one go were preferred. This is supported by findings from other studies on food choices, which showed that the subjective evaluation of food often occurs at the point of purchase or consumption^(27,40,41)^. The process involves a series of ‘criteria’, that is, how people perceive the value of a product based on its utility^(41)^.

Similar to the dynamic nature of personal factors, eating context was found to influence the portion size norms depending on where portion size selection took place and who was present. Portion size selection was dependent on where it took place and who was present. Examples included participants’ preferences for different sized packages of crisps for home settings when eating by themselves *v*. at social events, and serving sizes of cakes purchased at a coffee shop were dependent on whether friends or family members were present for sharing. Previous studies have proposed that norms of eating behaviours heavily relied on a desire for social approval, that is, people would replicate the behaviours of other people in the social group and how much they consume at one sitting^(2,3,16,42)^. We found that family influence was another important factor. Many participants noted that their norms of appropriate portion sizes were based on family education established in childhood and such norms were further modified depending on current living conditions (for example, influenced by partner’s family). In contrast, food environment factors were relatively more stable, reflecting external cues embedded in the broader food system such as the ranges of size options available at point-of-purchases, default sizes set by food outlets, promotion of different size options and serving size labelling^(4,43)^. The theme of ‘manufacturer power’ was consistently raised during all focus groups. It was recognised that food environment factors were determined by stakeholders including the food industry and government sectors rather than consumers themselves, which often restricted consumers from selecting portion sizes based on their preferences.

Furthermore, conceptualisations of norms of normal and appropriate portion sizes were distinguished. Normal portion sizes were regarded to be larger than appropriate portion sizes. Expectations of how much should be consumed to avoid potential negative consequences (for example, weight gain and health concerns from overconsumption) were frequently mentioned in discussions of appropriate portion sizes, whilst normal portion sizes appeared to be based on typical eating behaviours in day-to-day life^(2,3)^. This is consistent with a proposed theory that the norms of normal and appropriate portion sizes are not alternative terminologies^(1)^. Perceptions of normal portion sizes have not been specifically examined in the literature. Apart from the close relations to existing perceptions of appropriateness and social expectations^(1,3)^, norms of normal portion sizes appeared to be built upon a series of personal factors and internal cues of hunger, craving of palatable food, past experience and current lifestyle. In contrast, the perceptions of appropriate eating behaviours have a strong connection with the desire for social approval^(2,44)^. For discretionary food in particular, overconsumption is regarded as being indulgent, inappropriate and lacking in self-control^(45)^. This was consistent with findings of the current study where participants described their norm of appropriate portion sizes as ‘restricted’ and ‘in limited amounts’. Additionally, participants matched their habitual intake with norms of normal portion sizes and admitted to not following the norms of appropriate portion sizes. Potential reasons could be food palatability that overrides perceptions of appropriateness or overindulgence which may have become the new ‘social trend’ as discretionary foods are grossly overconsumed by the majority of the population^(2,46)^. It could also be in part because the appropriate portion sizes were presumed to be unacceptably small, thus an amount ‘slightly larger’ than that was considered more acceptable.

Consistent with a review on manufacturer power by academics in marketing^(47)^, our findings showed that food manufacturers had a substantial influence on serving size availability, consumers’ serving size purchase decisions and portion sizes consumed. The current serving sizes of cake and pastries were considered ‘too big’ by focus group participants, often with only one size option being available per food item. However, participants noticed the occurrence of ‘shrinkflation’ for several fast foods and confectionery, in which serving sizes had decreased over time whereas the price had remained the same or even increased. Better value for money and lower unit price of discretionary foods in larger serving sizes were consistently mentioned, which were believed to encourage overconsumption. This was attributed to the robust portion size effect^(42,43)^; participants noticed that larger serving sizes resulted in increased intake, and they would often consume more than intended to avoid food waste. Large serving sizes were, therefore, considered a major barrier to portion control. Environmental concerns were also raised as an increase in the availability of smaller serving sizes was believed to result in unnecessary packaging and plastic waste. Innovative and sustainable food packaging that allows for effective portion control would be beneficial; for instance, on-pack visual cues and resealable packages^(48,49)^. In addition, the current on-pack serving size information was deemed unhelpful and caused confusion. In the absence of guides around appropriate discretionary food portion sizes from public health sectors, the on-pack serving size information (the nutrition-information-panel) was commonly regarded as a recommendation. More serving size choices, changes to the sale strategy and proportional pricing between size options, as well as clearer serving size information and relevant recommendations are needed to allow consumers to have more control over their portion size selections^(25,40)^.

There are a few strengths of the methodologies used to complete this focus group study. An even number of males and females and participants living in both metro and rural areas were recruited. The semi-structured question guide was piloted in a target population to ensure usability. The inductive thematic analysis enabled generation of rich results from collected data. However, although the vast majority of the Australian population are active internet users^(50)^, the online design may have excluded those who were not computer literate or did not have access to the internet. We acknowledge that most participants were university-educated and lived in postcodes associated with higher socio-economic status. Careful interpretation of the results is required as other population samples may yield different findings, and the various needs of Australian consumers from different population subgroups and backgrounds should be considered. Additional studies need to be conducted with consumers from lower socio-economic status areas and those with limited access to healthy food choices to understand their opinions and behaviours.

### Conclusion

The framework shows the complex and dynamic nature of portion size norms for discretionary foods. This involves the interaction across various personal, eating context and food environment factors. Different factors were prioritised by individuals (e.g. quantity, quality or convenience) in conceptualising portion size norms and depended on eating context factors such as whether other people were present to share, as well as food environment factors such as the availability of options, unit price and sale strategies. The results also uncovered important distinctions between norms of normal and appropriate portion sizes, despite the awareness that normal portion sizes were larger than appropriate portion sizes, consumers noticed the tendency of consuming more than intended and generally failed to follow their norm of appropriate portion sizes.

From a public health perspective, our findings highlight that consumers had limited choices when selecting portion sizes in various settings and lacked the knowledge and skills in portion control. Potential strategies to help prevent the overconsumption of discretionary foods may include active engagement of the food industry to introduce a wider range of smaller serving sizes, in conjunction with proportional pricing, environmentally friendly packaging and increased efforts from public health authorities in providing guidance around appropriate portion sizes. Future investigations should explore these strategies in a variety of settings and assess the acceptability from the consumer’s perspective.
